# Dyadic Care Types and Quality of Life of patient-caregiver dyads in Multiple Chronic Conditions

**DOI:** 10.1093/geroni/igaf122.2933

**Published:** 2025-12-31

**Authors:** Maddalena De Maria, Jeffrey Stokes, Harleah Buck, Elliane Irani

**Affiliations:** Link Campus University, Rome, Lazio, Italy; University of Massachusetts Boston, Boston, Massachusetts, United States; University of Iowa, Iowa City, Iowa, United States; Case Western Reserve University, Cleveland, Ohio, United States

## Abstract

**Purpose:**

To examine how congruence in the appraisal of dyadic Multiple Chronic Condition (MCC) care type affects the quality of life (QOL) in patient-caregiver dyads for one year.

**Methods:**

Patient-caregiver dyads were recruited. Patients aged 65 years and older with a diagnosis of at least two chronic illnesses were enrolled. Those affected by cancer or dementia were excluded. Caregivers were eligible if they were identified by the patient as the primary caregiver. Patient and caregiver appraisal of dyadic care type was assessed using the Dyadic Symptom Management Type instrument. Congruence occurred when patients and caregivers agreed in their appraisal of who is responsible for MCC management. QOL was measured with the Short Form-12. Controlled longitudinal multilevel dyadic models were applied to assess how congruence in dyadic care type influenced both patients’ and caregivers’ mental and physical QOL over one year.

**Results:**

A convenience sample of 2,343 observations from 871 patient-caregiver dyads participated (mean age of 77 and 52 years, respectively). Both patient-oriented and collaborative-oriented congruence in care type were associated with higher levels of mental QOL for patients, while collaborative congruence was linked to higher levels of mental QOL for caregivers. In contrast, caregiver-oriented congruence was associated with lower Mental QOL for patients. For Physical QOL, patient-oriented congruence was associated with better and caregiver-oriented congruence with worse QOL for patients, whereas for caregivers only caregiver-oriented congruence was associated with worse QOL.

**Conclusion:**

Congruence itself was not a predictor of QOL, but rather the type of congruence played a key role.

